# Ecological functions of zoosporic hyperparasites

**DOI:** 10.3389/fmicb.2014.00244

**Published:** 2014-05-28

**Authors:** Frank H. Gleason, Osu Lilje, Agostina V. Marano, Télesphore Sime-Ngando, Brooke K. Sullivan, Martin Kirchmair, Sigrid Neuhauser

**Affiliations:** ^1^School of Biological Sciences A12, University of SydneySydney, NSW, Australia; ^2^Núcleo de Pesquisa em Micologia, Instituto de BotânicaSão Paulo, Brazil; ^3^Laboratoire Microorganismes: Génome and Environnement, Université Blaise Pascal, Clermont-Ferrand IIAubière, France; ^4^Back To Nature DesignSeattle, WA, USA; ^5^Institute of Microbiology, Leopold Franzens University InnsbruckInnsbruck, Austria; ^6^Microbial Diversity and Genomics, Department of Life Sciences, Natural History MuseumLondon, UK

**Keywords:** hyperparasites, ecology, food web, parasite, zoospores, eDNA

## Abstract

Zoosporic parasites have received increased attention during the last years, but it is still largely unnoted that these parasites can themselves be infected by hyperparasites. Some members of the Chytridiomycota, Blastocladiomycota, Cryptomycota, Hyphochytriomycota, Labyrinthulomycota, Oomycota, and Phytomyxea are hyperparasites of zoosporic hosts. Because of sometimes complex tripartite interactions between hyperparasite, their parasite-host, and the primary host, hyperparasites can be difficult to detect and monitor. Some of these hyperparasites use similar mechanisms as their parasite-hosts to find and infect their target and to access food resources. The life cycle of zoosporic hyperparasites is usually shorter than the life cycle of their hosts, so hyperparasites may accelerate the turnaround times of nutrients within the ecosystem. Hyperparasites may increase the complexity of food webs and play significant roles in regulating population sizes and population dynamics of their hosts. We suggest that hyperparasites lengthen food chains but can also play a role in conducting or suppressing diseases of animals, plants, or algae. Hyperparasites can significantly impact ecosystems in various ways, therefore it is important to increase our understanding about these cryptic and diverse organisms.

## Introduction

“*So, naturalists observe, a flea*

Has smaller fleas that on him prey;

*And these have smaller still to bite ‘em*,

And so proceed ad infinitum.”

Jonathan Swift, On Poetry: a rhapsody (1733)

Parasites belonging to all taxonomic groups have gained increasing attention in ecological research during recent years. It is widely recognised that the number of species of parasites are more numerous than organisms with a non-parasitic lifestyle (Lafferty et al., 2008). Also it is widely accepted that many parasites can themselves be hosts for other parasites. Such parasites of parasites are usually called “hyperparasites”; a term which is used without any reference to the phylogeny of the host or the parasite or whether the relationship is obligately or facultatively parasitic. Novel methodological tools and an increasing interest in parasites and their ecology have led to more targeted sampling approaches. This has shown that especially microbial parasites which have until now been rarely detected are abundant and diverse (Lefèvre et al., 2008; Jones et al., 2011; Hartikainen et al., 2014). It is very difficult—or in many cases impossible—to isolate and identify them because of their generic morphology, and because such parasites are often restricted to only a few host cells which makes them difficult to detect even with state of the art molecular methods. Hence, it is no surprise that microbial hyperparasites are not well understood. Some species of hyperparasites are endoparasites and difficult to see in the light microscope without special staining methods. Although zoosporic parasites of primary producers have been the focus of recent studies (Powell, 1993; Ibelings et al., 2004; Kagami et al., 2007; Marano et al., 2011; Neuhauser et al., 2011a), our knowledge about zoosporic hyperparasites and their microbial hosts remains anecdotal. In this article we focus on zoosporic hyperparasites with zoosporic hosts, their abundance and relationships between parasites and their hosts and their possible roles in ecological processes.

In two of the early works focusing on microbial hyperparasites, Karling (1942a,b) documented and discussed examples of hyperparasitism among zoosporic true fungi (Table 1). Although his study focused primarily on hyperparasites among the zoosporic true fungi, Karling was aware of hyperparasites among other microbial groups such as stramenopiles or plasmodiophorids (Table 2). Sparrow's monograph about aquatic phycomycetes contains still the most comprehensive references to zoosporic hyperparasites (Sparrow, 1960). Although hyperparasitism among true fungi has been the focus of numerous research projects, for instance in the form of biological control of plant diseases (e.g., Vinale et al., 2008), hyperparasitism involving heterotrophic stramenopiles and zoosporic true fungi has been rare (Boosalis, 1964; Barnett and Binder, 1973; Adams, 1990). Zoosporic hyperparasites have been described in the fungal groups Chytridiomycota, Blastocladiomycota, and Cryptomycota (Opisthokonts, for examples see Table 1). Within the heterokonts the groups Hyphochytriomycota, Oomycota, Labyrinthulomycota, and Phytomyxea contain hyperparasitic species (Table 2). These groups belong to various supergroups in the tree of life (Baldauf, 2003; Adl et al., 2012), but these microorganisms interact together in the same ecosystems. Because of their morphological similarity and their similarity in size they can have ecologically similar functions and are in food web studies often treated as “trophic species” (Powell, 1993; Marano et al., 2011). Many of the known hosts belong to common genera which are frequently observed in many soil and fresh water ecosystems using both baiting procedures and molecular analysis of environmental samples (Sparrow, 1960; Powell, 1993; Barr, 2001; Dick, 2001; Lozupone and Klein, 2002; Shearer et al., 2007; Lefèvre et al., 2008; Marano et al., 2011). It is very likely that zoosporic hyperparasites are as abundant on “rarer” hosts. This is of ecological importance because zoosporic true fungi and heterotrophic stramenopiles can be among the predominant groups in some ecosystems (Lefèvre et al., 2008; Freeman et al., 2009; Marano et al., 2011). Because of the large number of species of zoosporic parasites, hyperparasites, and their associated hosts, it is likely that there are many additional taxa that await discovery.

**Table 1:** **Selected hyperparasitic Opistokonts (Chytridiomycota, Cryptomycota, Blastocladiomycota)**.

**Hyperparasite**	**Trophic mode**	**Parasite (=Host of hyperparasite)**	**Host (=Host of parasite)**	**References**
**Cryptomycota**		**Chytridiomycota**		
*Rozella marina*	Biotroph	*Chytridium polysiphoniae*	Parasite, red algae	Sparrow, 1960; Held, 1981
*Rozella parva*	Biotroph	*Zygorhizidium affluens*		Canter, 1965; Beakes et al., 1988
*Rozella rhizophlyctii*	Biotroph	*Rhizophlyctis rosea*	Facultative parasite	Karling, 1960; Held, 1981
	Biotroph	*Rhizophydium globosum*	Parasite, Diatoms, algae	Sparrow, 1960; Held, 1981
*Rozella polyphagi*	Biotroph	*Polyphagus laevis*	Parasite, *Euglena*	Sparrow, 1960; Held, 1981
	Biotroph	*Polyphagus euglenae*	Parasite, *Euglena*	Powell, 1984
*Rozella endochytrium*	Biotroph	*Endochytrium operculatum*	Facultative parasite, algae	Sparrow, 1960; Held, 1981
*Rozella cladochytrii*	Biotroph	*Cladochytrium replicatum*	Facultative parasite, green algae	Sparrow, 1960; Held, 1981
**Cryptomycota**		**Blastocladiomycota**		
*Rozella allomycis*	Biotroph	*Allomyces arbuscula*	Facultative parasite, insect cadaver	Held, 1981
	Biotroph	*Allomyces macrogynus*		Held, 1974
**Cryptomycota**		**Oomycota**		
*Rozella rhipidii-spinosi*	Biotroph	*Araiospora spinosa*	Facultative parasite	Sparrow, 1960; Held, 1981
*Rozella apodiae-brachynematis*	Biotroph	*Apodachlya brachynema*	Facultative parasite	Sparrow, 1960; Held, 1981
*Rozella achlyae*	Biotroph	*Achlya flagellata*	Facultative parasite	Sparrow, 1960; Held, 1981
		*Dictyuchus anomalus*	Parasite, fish	
*Rozella cuculus*	Biotroph	*Pythium intermedium*	Parasite, plant	Sparrow, 1960; Held, 1981
		*P. monospermum*	Parasite, nematode	Held, 1981
*Rozella laevis*	Biotroph	*Pythium gracile*	Parasite, green algae	Sparrow, 1960; Held, 1981
*Rozella barrettii*	Biotroph	*Phytophthora cactorum*	Parasite, plant	Sparrow, 1960; Held, 1981
*Rozella pseudomorpha*	Biotroph	*Lagenidium rabenhorstii*	Parasite, green algae	Sparrow, 1960; Held, 1981
**Chytridiomycota**		**Chytridiomycota**		
*Dictyomorpha dioica*	Biotroph	*Achlya flagellata*		Mullins and Barksdale, 1965
Chytridium parasiticum	Biotroph	*Septosperma rhizophydii*	Parasite, chytrid	Karling, 1960
*Rhizophydium parasiticum*		*Rhizophlyctis rosea*	Facultative parasite, chitin	Karling, 1960; Sparrow, 1960
		*Chytridiomyces verrucocsa*		
*Rhizophydium carpophilum*		*Synchytrium fulgens*	Parasite, plant	Karling, 1960
		*S. macrosporum*	Parasite, plant	
		*S. linariae*	Parasite, plant	
*Phlyctochytrium synchytrii*		*Synchytrium endobioticum*	Parasite, plant	Karling, 1942a
*Septosperma rhizophydii*		*Rhizophydium macrosporum*	Facultative parasite	Karling, 1960
*Septosperma anomala*		*Phlyctidium bumelleriae*	Parasite, Xanthophyceae	Karling, 1960
**Chytridiomycota**		**Oomycota**		
*Rhizophydium pythii*	Biotroph	*Pythium monospermum*	Parasite, nematode	Sparrow, 1960
*Rhizidiomyces japonicus*		*Phytophthora megasperma*	Parasite, plant	Sneh et al., 1977
		*Phytophthora erythroseptica*	Parasite, plant	Wynn and Epton, 1979
*Canteriomyces stigeoclonii*		*Phytophthora megasperma*	Parasite, plant	Sneh et al., 1977
**Blastocladiomycota**		**Oomycota**		
*Catenaria anguillulae*	Facultative	*Phytophthora cinnamomii*	Parasite, plant	Daft and Tsao, 1984
		*Phytophthora parasitica*	Parasite, plant	

*Hyperparasites and hosts are sorted by taxon. Higher ranks are given in bold*.

**Table 2:** **Selected hyperparasitic Heterokonts (Oomycota, Hyphochytridiomycota, Phytomyxea)**.

**Hyperparasite**	**Trophic mode**	**Parasite (=Host of hyperparasite)**	**Host (=Host of parasite)**	**References**
**Oomycota**		**Oomycota**		
*Olpidiopsis incrassata*		*Saprolegnia ferax*	Parasite, fish	Slifkin, 1961
*Olpidiopsis karlingiae*		*Rhizophlyctis rosea*	Facultative Parasite	Karling, 1960
*Pythiella vernalis*		*Pythium aphanidermatum*	Parasite, plant	Pires-Zottarelli et al., 2009
		*Pythium gracile*	Parasite, green algae	Blackwell, 2010
*Pythiella pythii*		*Pythium dictyosporum*	Parasite, green algae	Blackwell, 2010
*Pythium proliferum*		*Rhizophlyctis rosea*	Facultative Parasite	Karling, 1960
*Pythium monospermum*		*Phytophthora megasperma*	Parasite, plant	Humble and Lockwood, 1981
*Pythium oligandrum*		*Pythium irregulare*	Parasite, plant	Ribeiro and Butler, 1995; Benhamou et al., 1999
		*Pythium mamillatum*	Parasite, plant	
		*Pythium paroecandrum*	Parasite, plant	
		*Pythium aphanidermatum*	Parasite, plant	
		*Pythium sylvaticum*	Parasite, plant	
		*Pythium ultimum*	Parasite, plant	
**Hyphochytridiomycota**		**Oomycota**		
*Hyphochytrium catenoides*	Facultative	*Pythium myriostylum*	Parasite, plant	Ayers and Lumsden, 1977
		*Aphanomyces euteiches*	Parasite, plant	Ayers and Lumsden, 1977; Sneh et al., 1977
		*Phytophthora erythroseptica*	Parasite, plant	Wynn and Epton, 1979
		*Phytophthora megasperma*	Parasite, plant	Humble and Lockwood, 1981
**Phytomyxea**		**Oomycota**		
*Sorodiscus cokeri*	Biotroph	*Pythium proliferum*	Facultative Parasite	Goldie-Smith, 1951
		*Pythium graminicolum*	Facultative Parasite, moss	Goldie-Smith, 1951
		*Pythium catenulatum*	Facultative Parasite, plant	Goldie-Smith, 1951
		*Pythium elongatum*	Facultative Parasite	Goldie-Smith, 1951
		*Pythium irregulare*	Parasite, plant	Goldie-Smith, 1951
		*Pythium undulatum*	Parasite, plant	Goldie-Smith, 1951
*Woronina polycystis*	Biotroph	*Saprolegnia ferax*	Parasite, fish	Goldie-Smith, 1954
*Woronina pythii*	Biotroph	*Pythium proliferum*	Facultative Parasite	Goldie-Smith, 1956a
		*Pythium aphanidermatum*	Parasite, plant	Goldie-Smith, 1956a
		*Pythium debaryanum*	Parasite, plant	Goldie-Smith, 1956a
		*Pythium irregulare*	Parasite, plant	Goldie-Smith, 1956a
		*Pythium monospermum*	Parasite, nematode	Goldie-Smith, 1956a
		*Pythium pulchrum*		Goldie-Smith, 1956a
		*Pythium ultimum*	Parasite, plant	Goldie-Smith, 1956a

*Hyperparasites and hosts are sorted by taxon. Higher ranks are given in bold*.

## Zoospores

Zoospores are a shared morphological feature of the hosts and hyperparasites discussed here. Zoospores are motile propagules which permit rapid dispersal. Zoospores can sense environmental gradients which they use to identify and find potential hosts (Tyler, 2002). There are different types of zoospores (Lange and Olson, 1983), which have distinguishing features, allowing observers to determine and categorize the organisms. The most important feature is the type of flagellation. Zoospores can generally be grouped into (1) uniflagellate with posteriorly directed whiplash flagellum, (2) uniflagellate with an anteriorly directed tinsel flagellum, (3) biflagellate, heterokont, with one posteriorly directed whiplash flagellum and one anteriorly directed tinsel flagellum and (4) biflagellate, isokont, two whiplash flagellae, often of different lengths, with the shorter one anteriorly directed and the longer one posteriorly directed.

Despite their relatively simple morphology many zoosporic hyperparasites form functionally and developmentally distinct types of zoospores during their life cycle (Sparrow, 1960). A variety of names are used for different types of zoospores in different taxonomic groups, but generally one type of zoospore is formed in zoosporangia following mitosis and can be either haploid or diploid, while another type of zoospore is formed by meiosis and is haploid (Lange and Olson, 1983). The different types of zoospores can serve different functions during the parasite life cycle—such as rapid propagation and dispersal or primary infection and population establishment after periods of hibernation (e.g., Neuhauser et al., 2011b). Despite variable modes of formation and complex parasite life cycles which can result in periods where one type of zoospore is predominantly formed, the main unifying feature of all types of zoospores is that they are small, single-celled, motile propagules. Within food webs zoospores provide a rapid energy source for a variety of organisms at higher trophic levels (Gleason et al., 2011), so it is not surprising that zoospores are often treated as trophic species.

## Mechanisms used by hyperparasites to access food resources

**Figure 1:**
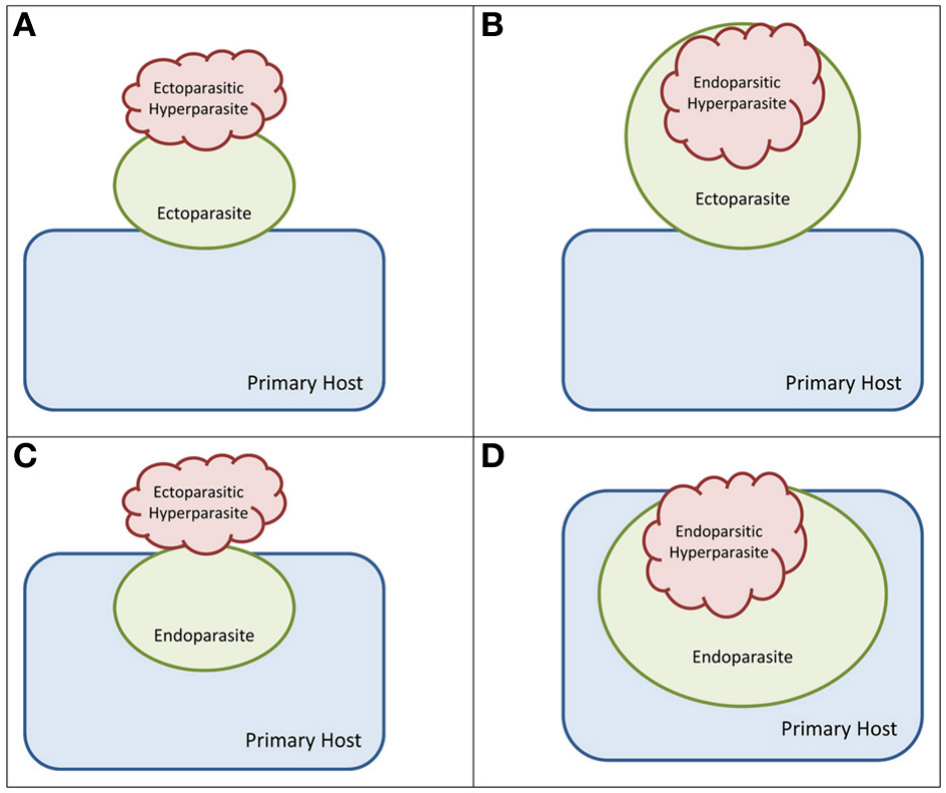
**Types of hyperparasitism**. Blue—primary host, green—parasite, red—hyperparasite. **(A)** epibiotic hyperparasite of ectoparasite. This type can be found for example in the interaction of the hyperparasite *Rhizophydium parasiticum* (Chytridiomycota), its and its (facultative) parasites host *Rhizophlyctis rosea*. **(B)** Endobiotic hyperparasite of ectoparasite host. This is the most commonly described mode of hyperparasitsm seen in many *Rozella* species (Cryptomycota) or *Woronina* spp. (Phytomyxea). **(C)** Epibiotic hyperparasite of endoparasite host. E.g., *Rhizophyidum carpophilum* (Chytridiomycota) on *Olpidiopsis* sp. (oomycetes) and *Synchytrium* sp. (chytrid). **(D)** Endobiotic hyperparasite of endoparasite host. E.g., the hyperparasitic chytrid *Phlyctochytrium synchytrii* in the plant pathogen *Synchytrium endobioticum*.

Zoosporic hyperparasites use a large variety of mechanisms to attack their hosts. Hyperparasites can grow epibiotically on the surface of their host only entering the host cell with specialized structures such as chytrid rhizoids (Figures 1A,C). Hyperparasites also grow endobiotically this means completely submerged in their hosts (Figures 1B,D). The parasite hosts of hyperparasites can be ectoparasites (Figures 1A,B) growing epibiotically on the primary host or endoparasites (Figures 1C,D) growing endobiotically inside the primary host. Hyperparasites which are infecting ectoparasites only have to overcome the defense mechanisms of their host, and often use infection strategies that are very similar to those of zoosporic parasites (Sparrow, 1960; Marano et al., 2012). On the other hand, hyperparasites which are parasites of endoparasites may have to overcome two barriers of defense—they have to enter the parasite-host and their host to get access to food resources. Most of the described zoosporic hyperparasites are parasites of ectoparasites (e.g., most *Rozella* species, *Wornina* spp.). We hypothesize that ectoparasites are easier accessible for hyperparasites with only one line of defense to break. We also hypothesize that our knowledge about zoosporic hyperparasites of endoparasites is biased by the fact that zoosporic endoparasites are a poorly studied group themselves. Therefore, most of the examples discussed here are from zoosporic hyperparasites of parasites which are not completely submerged inside their host or from endoparasitic hyperparasites of epibiotic hosts (Figures 1A–C).

An example of an epibiotic infection (Figure 1A) is the parasitic relationship between the two chytrids *Chytriomyces verrucosus* and *Rhizophlyctis rosea* (Karling, 1960). The chemotactic zoospores of *R. rosea* are attracted to the host cell where they encyst. The zoospore then germinates and a germ tube penetrates the host zoosporangium. Inside the host, an endobiotic rhizoidal system develops supplying the epibiotic zoosporangium (having since formed from the body of the zoospore) with nutrients. Epibiotic parasites can also be found in the stramenopiles (Sneh et al., 1977): zoospores of the hyphochytriomycete *Rhizidomyces japonicus* attach to the surface of oospores of *Phytophthora megasperma* (Oomycetes) where thalli grow externally around the oospore and produce zoosporangia. The oomycete *Pontisma lagenidioides* which is a parasite of the green alga *Chaetomorpha media* can be infected by *Labyrinthula* sp. (Raghukumar, 1987).

Endobiotic parasites grow entirely submerged within their host. An example is *Rozella allomycis* (Rozellida/Cryptomycota) and its host *Allomyces arbuscula* (Blastocladiomycota) (Held, 1973, 1974). In this case, the infection process is relatively well studied and is described in more detail here to exemplify the infection process of most known endobiotic zoosporic hyperparasites. Substances produced by the host attract the chemotactic zoospores of the parasite toward the host. Once the zoospore attaches to the surface of the host cell it forms a so-called cyst, which produces a germ tube. The germ tube then grows into the host cell through the cell wall while the protoplast of *Rozella* is pushed into the host cell by fluid pressure produced from a vacuole in the cyst. Subsequently the parasite grows inside the host cell. In the case of *Rozella allomycis* the host cell is then transformed into the parasite sporangium. Other known endobiotic parasites are *Rozella polyphagi* (Rozellida/Cryptomycota), which parasitizes the chytrid parasite *Polyphagus euglenae* (Powell, 1984) and the endobiotic parasite *Catenaria allomycis* (Blastocladiomycota), which infects *Allomyces javanicus* (Sykes and Porter, 1980; Powell, 1982). *Catenaria anguillulae*, a member of the Blastocladiomycota, is an endobiotic parasite of the plant pathogenic oomycetes *Phytophthora cinnamomi* and *P. parasitica* (Daft and Tsao, 1984), while *Hyphochytrium catenoides* (Hyphochytriomycota) colonizes oospores of *Pythium myriostylum* (Ayers and Lumsden, 1977). Another parasite of *Pythium* spp. is *Woronina pythii* (Phytomyxea), which infects both vegetative hyphae and reproductive structures of *Pythium* (Dylewski and Miller, 1983).

Interactions are slightly different between hyphal forming zoosporic organisms, such as oomycetes. Here interactions between hyphae can be observed, and these interactions are different from the endo- and epibiotic parasitic interactions discussed above. Two distinct mechanisms appear to be involved in interactions between this parasite and its hosts: (1) hyperparasitism; mediated by hyphal interactions, and (2) antibiosis; causing metabolic and developmental changes prior to contact between hyphae of the parasite and host (Adams, 1990; Benhamou et al., 1999). An example of direct interactions between the organisms is the interaction between hyphae of the well-known hyperparasite *Pythium oligandrum* (Oomycota) and hyphae of its oomycete hosts (e.g., *P. ultimum, P. aphanidermatum, Phytophthora megasperma*) (Benhamou et al., 1999). Hyphae of the parasite can adhere to the surface of the host sometimes coiling around the host hyphae. Penetration of the host cells by infection pegs may follow, leading to digestion of the host cytoplasm. When the interaction is initiated by antibiosis (without contact with the host) the parasite can release soluble substances which cause biochemical changes within the host cells. Then the parasite can release extracellular enzymes, which digest the host cells.

## Biodiversity and host range of hyperparasites

DNA sequences assigned to putative parasite and hyperparasite taxa of zoosporic fungi are widespread (e.g., Lara et al., 2010; Jones et al., 2011; Lara and Belbahri, 2011; Nagano and Nagahama, 2012). But molecular methods are often biased by the selection of primers and sampling methods (Hartikainen et al., 2014; Neuhauser et al., 2014) and the assignment of environmental DNA sequences to described species is only as good as the available reference datasets. Data on zoosporic microorganisms are sparse, and many of the “unknown” sequences are probably from common species which to date have no reference record in public data bases (e.g., Nagy et al., 2011; Karpov et al., 2013). Reliable reference sequences of many zoosporic hyperparasites are generally rare. One reason is that many of the known zoosporic hyperparasites are biotrophic parasites which cannot be grown without their hosts. The hosts themselves are often biotrophic parasites as well, making it very hard to isolate, identify and sequence the hyperparasites. Therefore, targeted studies to detect and characterize hyperparasites and their hosts are needed. Such targeted approaches could include baiting experiments combined with microscopic observation or DNA and RNA based screenings of various environments. Despite being very time consuming baiting and isolation experiments are highly valuable because they will allow to understand how hyperparasites interact with their hosts, to describe their life cycle, and to analyze interactions with their hosts. Baiting experiments with oospores of the oomycetes parasites *Phytophthora megasperma, P. cactorum, Pythium* sp. and *Aphanomyces euteiches*, revealed that those baits quickly became infected by different hyperparasites (Sneh et al., 1977). Another approach for characterizing zoosporic hyperparasites would be to implement a combination of DNA and RNA isolation methods combined with specific primers and to then visualize the respective organisms using specific FISH (Fluorescence *in situ* hybridization) probes (Not et al., 2002; Jones et al., 2011; Marano et al., 2012). Such targeted molecular probing techniques are a powerful tool to identify unknown organisms. When attempting to detect hyperparasites by this approach, however, mainly free living stages (zoospores) will be detected and the sampling is largely limited to aquatic environments because the background fluorescence in soil or sediment samples tends to be high (Wagner and Haider, 2012).

Hyperparasites, their hosts and the primary hosts are complex systems. Most studies about zoosporic hyperparasites base their evidence on laboratory studies of dual cultures of one host infected by one parasite or the host range of a single parasite (e.g., Karling, 1960; Sparrow, 1960; Held, 1981). Although to date we can only estimate how those interactions might occur in natural environments like sediment or soil (Gleason et al., 2012), simultaneous infections by different species are likely—especially for abundant parasite hosts for which more than one species of hyperparasite is known (for examples see Tables 1, 2). Similarly, unrelated or distantly related hyperparasites may infect the same hosts individually or simultaneously. An excellent example of this phenomenon was described by Karling (1960) who observed simultaneous infection of *Rhizophlyctis rosea* with four hyperparasites. He studied infections of the facultative parasite *R. rosea* with *Chytriomyces verrucosa* (Chytridiomycota). Karling noted that numerous sporangia of *R. rosea* were also infected with *Rozella rhizophlyctii* (Rozellida/Cryptomycota) and *Olpidiopsis karlingiae* (Oomycota). In addition to this, the large sporangia of *R. rosea* were infected by a fourth species, *Pythium proliferum* (Oomycota), which was itself densely parasitized by *Woronina pythii* (Phytomyxea). Although *R. rosea* is a facultative parasite, this example shows the extent to which hyperparasites can occur in nature when studied in detail.

On the other hand not all hyperparasites are host specific. Studies on the range of host specificity indicate that some species of hyperparasites in the Oomycota and Phytomyxea can infect several species of hosts (Goldie-Smith, 1951; Dylewski and Miller, 1983). *Rozella allomycis* only infects two susceptible hosts: *Allomyces arbuscula* and *A. macrogynus* (Held, 1974), while *Olpidiopsis incrassata* infects six species of *Saprolegnia* and three species of *Isoachlya* (Slifkin, 1961). Other parasites such as *Woronina pythii* have a broad host spectrum and can infect more than 40 species of oomycetes (Dylewski and Miller, 1983). *Pythium oligandrum* also infects a wide range of fungal and stramenopilous host (Ribeiro and Butler, 1995). These studies highlight the importance of isolating and characterizing species for understanding and characterizing hyperparsite biodiversity and host range. Culture based methods and well defined voucher isolates are also needed to provide a groundwork for DNA barcoding studies (del Campo et al., 2014) or for food web analyses (Hrcek et al., 2011) which form the basis for a more holistic understanding of hyperparasites and their ecological roles.

## Size control of host populations by hyperparasites

Like all parasites, hyperparasites can impact population size and fitness of their hosts (Sieber and Hilker, 2011; Allen and Bokil, 2012; Preston et al., 2014). Some hyperparasites can infect persistent structures of their hosts, for example oospores, resistant sporangia, or resting spores (Gleason et al., 2010). Such resting stages are recalcitrant substrates and can survive in a dormant state in dried soil for long periods of time (Goldie-Smith, 1956b; Bruckart et al., 2011) where they accumulate, forming a “spore bank” of zoosporic parasites. But when these resting stages are infected by hyperparasites the pathogen pressure can potentially be reduced. This could explain the finding that zoosporic hyperparasites can be linked to suppressive soil properties (Weller et al., 2002) as they have the ability to reduce the viable pathogen load in soil. The presence of hyperparasites contributes to controlling their hosts in the environment, hinting at the important role of these parasites in balancing diversity and abundance of their hosts, consequently resulting in stable ecosystems.

Hyperparasites are already widely used as biological control agents to control the population size of plant pathogens. The best known example is the oomycete *Pythium oligandrum* which is used to control other *Pythium* spp. and oomycetes (Ikeda et al., 2012). Hyperparasites have a huge potential to control diseases if they can be systematically accumulated in the environment. But so far not many hyperparasites can be grown in the lab in big enough quantities that permit use as biocontrol agent. There are known hyperparasites of important plant pathogens which have not been explored as biocontrol agents because of this reason. Oospores of the potato pathogen *Phytophthora erythroseptica*, for example, were found to be infected with *Hyphochytriun catenoides* and *Rhizidiomyces japonicus* in waterlogged soils in England (Wynn and Epton, 1979). Given the global importance of *Phytophthora* spp. as existing and emerging plant pathogens (Brasier et al., 2004; Fry, 2008; Fisher et al., 2012), identifying hyperparasites that naturally control the abundance and survival of these parasites would be beneficial.

There have been observations of such effects in control of population sizes by hyperparasites in fresh water ecosystems. Populations of *Zygorhizidium affluens* (Chytridiomycota) are frequent parasites of populations of the diatom *Asterionella formosa* in freshwater lakes (Canter, 1965; Beakes et al., 1988). The growth of the parasite population follows the growth of the host population (Chave, 2013) resulting in a “chytrid epidemic.” Sporangia and resting spores of *Z. affluens* can be infected by the hyperparasite *Rozella parva* (Canter, 1965). Both a decline in the *A. formosa* populations and an increase in the *R. parva* populations as the growing season progresses would, in theory, result in a decrease in *Z. affluens* populations. Another example is *Polyphagus euglenae*, a parasite of *Euglena viridis* and *E. gracilis* and its hyperparasite *Rozella polyphagi* (Powell, 1984), in which an infection with the hyperparasite *R. polyphagi* is known to decrease the population size of its host. Blooms of toxic cyanobacteria are common in freshwater environments (Sønstebø and Rohrlack, 2011). These cyanobacteria can be parasitized by zoosporic true fungi (Canter, 1972) that have the potential to control the sizes of such toxic algal blooms. Parasites of cyanobacteria can be infected by hyperparasites, a fact which was noted, but not analyzed in any detail. A reduction in the numbers of zoosporic parasites may result in an increase in growth of the (toxic) algal blooms (Canter, 1972). However, such tripartite interactions should be the subject of future studies: hyperparasites may impact the population sizes of parasitic, zoosporic true fungi that are parasites of organisms which can be damaging to the environment. The need to study the ecological role of hyperparasites may be even more significant as cyanobacteria and microalgae are gaining increasing importance as sustainable second generation biofuels (Stephens et al., 2010). Microalgal cultures are prone to get contaminated with a wide range of bacteria and eukaryotes which potentially impact on the yield (Stephens et al., 2010; Lakaniemi et al., 2012). Especially in such semi-controlled systems a control of detrimental parasites with hyperparasites could be a successful approach to increase productivity and energy yield.

## Food webs

**Figure 2:**
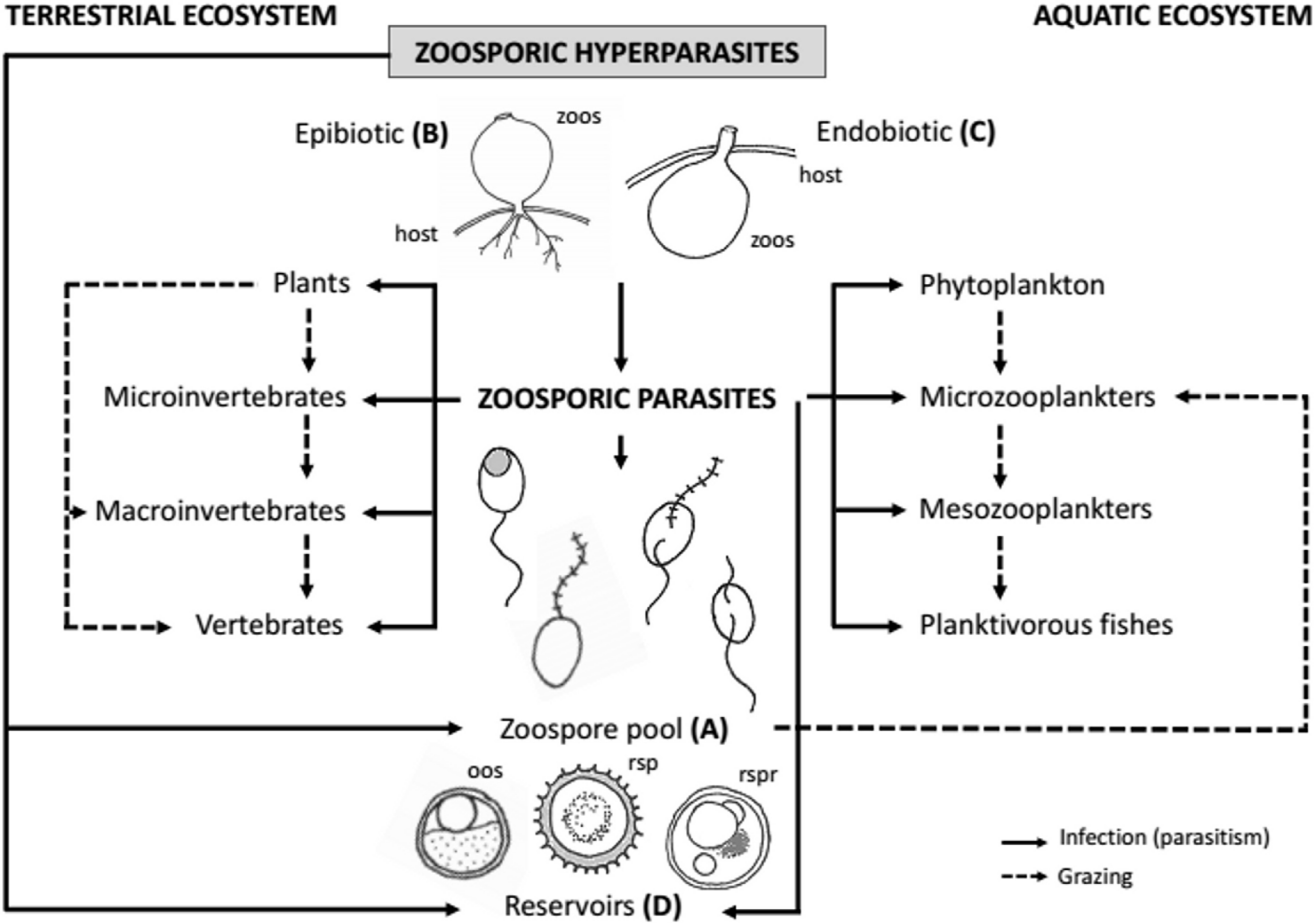
**Possible links of a hypothesized food web in which zoosporic parasites and hyperparasites are involved**. In food webs zoosporic hyperparasites can either contribute the zoospore pool (Zoospore pool, **A**) which is used as food source by grazers in terrestrial and acquatic ecosystems. At the same time epibiotic sporangia of hyperparasites (Epibiotic, **B**) can serve as food source for larger grazers. The sporangia of epibiotic hyperparasites (Endobiotic, **C**) are more difficult to access as food sources for grazers. Some zoosporic hyperparasites use resting stages (Reservoirs, **D**) as substrate. Hosts of hyperparasites can be parasites of microscopic eukaryotes, but also parasites of plants or animals. This allows for a rapid cycling of nutrients from organisms higher up in the food web towards small grazers (trophic upgrading). References: zoos, zoosporangium; host, zoosporic host; oos, oospore; rsp, resting spore; rspr, resting sporangium.

The presence of hyperparasites in food webs affect predators and grazers alike (Figure 2) (Hatcher et al., 2006; Morozova et al., 2007). By infecting resistant structures of their hosts, zoosporic parasites and hyperparasites release recalcitrant carbon, which is then potentially made available as food for protistan and metazoan predators rather than being deposited through sedimentation (Figure 2D). When zoospores are released, some will find new utilizable substrates, some will encyst, but many may provide food for grazing zooplankton and filter feeding animals (Figure 2A) (Kagami et al., 2007; Miki et al., 2011). The sizes of the mouth parts of grazing zooplankters determines the maximum size of zoopores that can be ingested (Kagami et al., 2007). For example, species of *Daphnia* are known to digest zoospores of any species smaller than 5 μm in diameter. The sizes of zoospores of hyperparasites tend to be smaller than those of the hosts (Sparrow, 1960; Held, 1981). This is clearly exemplified by the parasitic relationship between the fish parasite *Achlya flagellata* and its hyperparasite *Dictyomorpha dioica* (Mullins and Barksdale, 1965). The zoospores of *A. flagellata* are 8.5–10.5 μm in diameter while those of *D. dioica* are 3.5 μm in diameter (Mullins and Barksdale, 1965). The smaller size of the hyperparasite zoospores may enable zooplankton to graze on them or make their ingestion by zooplankters more likely, so that they ultimately provide better food resources for zooplankton than parasite zoospores. The population sizes of key species of grazing zooplankters, such as *Daphnia*, may be impacted by a decrease or increase in the total supply of zoospores which are a good food source (Kagami et al., 2007). This in turn will impact the population sizes of planktonivorous fish and other macroinvertebrates which feed on zooplankton.

Because of the high nutritional value of zoospores, we would expect populations of *Daphnia magna* to increase with the onset of the chytrid epidemic. *Daphnia magna* also feeds on zoospores of *Batrachochytrium dendrobatidis* (Chytridiomycota), which is a serious pathogen of amphibians (Buck et al., 2011). It was suggested that the consumption of zoospores of *B. dendrobatidis* by *D. magna* may prevent the transmission of this fungus (Buck et al., 2011). If a crash occurs in populations of *D. magna*, when the total zoospore food supply rapidly decreases, the rate of transmission of amphibian chytridiomycosis could increase because fewer individuals of *D. magna* would be present to feed on zoospores of *B. dendrobatidis*. Thus, more zoospores would be available to spread chytridiomycosis through the populations of amphibians. In adult frogs *B. dendrobatidis* prevalence is highest during late summer and winter, while infection takes place from late spring to early summer (Russell et al., 2010; Sapsford et al., 2013). This coincides with the breakdown of the chytrid epidemics. We would expect many other biotic and abiotic factors to affect population dynamics here, but the availability of zoospores as food in the spring can be decisive for the pathogen load of *B. dendrobatidis* later in the year by influencing the numbers of predators feeding on zoospores.

It is important to establish the roles of zoosporic hyperparasites as well as parasites in the structure and function of aquatic food webs. Structure includes species richness, trophic levels, links, trophic chain length, and connectance (Dunne et al., 2005, 2013). Function includes the total amount, rate, and efficiency of carbon transfer, and effects on stability of the food web. Adding parasites to food webs results in an increased complexity (Lafferty et al., 2008; Thieltges et al., 2013). Adding links to food webs, such as parasites, hyperparasites, and both of their associated niches, might also add to the stability of a particular web (Hudson et al., 2006; Lafferty et al., 2006, 2008). Parasites with life cycles involving ontogenetic niche shifts—such as hyperparasites—impact food web structures more and potentially negatively because specialized life cycle stages are more prone to secondary extinction than generalist stages (Preston et al., 2014). Such ontogenetic effects can be found in zoosporic hyperparasites: different types of zoospores, or zoospores formed by different species can have considerably different swimming patterns (Lange and Olson, 1983) or serve different purposes like long or short distance dispersal (Neuhauser et al., 2011a). Consequently different zoospores will attract predators occupying different niches and will therefore enter the food web at different trophic levels. Because of the anecdotal nature of the available data it is not yet possible to include zoosporic hyperparasites into mathematical food web models to allow for more realistic estimates of population dynamics and energy flow and their impact on food web stability. However, it can be expected that once our knowledge about zoosporic hyperparasites increases, we will also be able to show that, like zoosporic true fungi, zoosporic hyperparasites are diverse, abundant, and important links for energy transfer (Grami et al., 2011; Niquil et al., 2011). Zoosporic true fungal parasites result in a significant reduction in the loss of algal carbon though sedimentation into the detritus pool, allowing carbon transfer from zoospores to grazing protists and metazoans. This contributes to longer carbon path lengths, higher levels of activity and specialization, lower recycling, and increased stability of aquatic food webs (Grami et al., 2011; Ulanowicz et al., 2014).

Hyperparasites tend to have shorter life cycles than their hosts, so they produce biomass in the form of zoospores more quickly. Some of them produce primarily zoospores, such as *Rozella*, which, instead of forming its own zoosporangium, uses the host sporangium to reproduce (Held, 1981; Powell, 1984). This outsourcing of energy consuming biomass production allows for faster life cycles and hyperparasites such as *Rozella* are therefore likely to increase and accelerate the energy flow between trophic levels (Figure 2C). On the other hand epibiotic parasites have zoosporangia that are formed on the surface of their host. Consequently, both their zoospores and the zoosporangia are likely to enter the food web contributing different types of energy for predators with different size preferences for their food (Figure 2B). Since food webs that include zoosporic hyperparasites have additional links, we suggest they could be more efficient, and therefore would support a larger population of grazing zooplankton species. This hypotheses needs to be tested quantitatively.

## Conclusion and future prospects

Many hyperparasites have been discovered during research with the host species. However, it is vital that such efforts are intensified to provide the basis for the development of more rapid tools for species discovery and characterization. Although emerging techniques such as single cell genomic approaches provide a quantum leap in identifying and characterizing active cells in the environment, such methods will initially not account for the complex life cycles of zoosporic hyperparasites. To understand the life cycles, and consequently the ecological function of hyperparasites, time consuming studies involving targeted sampling and probing approaches are still needed. Even the sparse information available on hyperparasites highlights their potential in many ecosystem processes. Zoosporic hyperparasites may increase the turn-around time of certain nutrients in food webs due to their often rapid life cycles. They may play a role in trophic upgrading, as well as in the stability and complexity of food web dynamics. Hyperparasites also may play a role in the natural regulation of their host population sizes, which are also parasites. Regulation of population sizes of parasites will have an impact on their host population sizes. This may result in fine-tuning the magnitudes of patterns of energy flow in food webs and impact overall biodiversity as well as population dynamics. In summary, it is likely that zoosporic hyperparasites play a vital part of every ecosystem; hence more focused research on these important organisms is needed.

## Author contributions

Frank H. Gleason and Sigrid Neuhauser drafted the initial version of the manuscript. Agostina V. Marano, Télesphore Sime-Ngando, Martin Kirchmair, Brooke K. Sullivan and Osu Lilje critically revised this draft and contributed intellectual content to the final version.

### Conflict of interest statement

The reviewer, Hicham El Alaoui, declares that despite being affiliated to the same institution and department as the author, Télesphore Sime-Ndando, the review process was handled objectively and no conflict of interest exists. The authors declare that the research was conducted in the absence of any commercial or financial relationships that could be construed as a potential conflict of interest.
